# Use of patellofemoral digital twins for patellar tracking and treatment prediction: comparison of 3D models and contact detection algorithms

**DOI:** 10.3389/fbioe.2024.1347720

**Published:** 2024-02-23

**Authors:** Florian Michaud, Alberto Luaces, Francisco Mouzo, Javier Cuadrado

**Affiliations:** Laboratory of Mechanical Engineering, CITENI, Campus Industrial de Ferrol, University of La Coruña, Ferrol, Spain

**Keywords:** multibody dynamics, contact detection, contact forces, digital twin, total knee replacement, patellar tracking, simulation, treatment prediction

## Abstract

**Introduction:** Poor patellar tracking can result in painful contact pressures, patella subluxation, or dislocation. The use of musculoskeletal models and simulations in orthopedic surgeries allows for objective predictions of post-treatment function, empowering clinicians to explore diverse treatment options for patients. Although a promising approach for managing knee surgeries, the high computational cost of the Finite Element Method hampers its clinical usability. In anticipation of minimal elastic deformations in the involved bodies, the exploration of the Multibody Dynamics approach emerged as a viable solution, providing a computationally efficient methodology to address clinical concerns related to the knee joint.

**Methods:** This work, with a focus on high-performance computing, achieved the simulation of the patellofemoral joint through rigid-body multibody dynamics formulations. A comparison was made between two collision detection algorithms employed in the simulation of contact between the patellar and femoral implants: a generic mesh-to-mesh collision detection algorithm, which identifies potential collisions between bodies by checking for proximity or overlap between their discretized mesh surface elements, and an analytical contact algorithm, which uses a mathematical model to provide closed-form solutions for specific contact problems, but cannot handle arbitrary geometries. In addition, different digital twins (3D model geometries) of the femoral implant were compared.

**Results:** Computational efficiency was considered, and histories of position, orientation, and contact force of the patella during the motion were compared with experimental measurements obtained from a sensorized 3D-printed test bench under pathological and treatment scenarios. The best results were achieved through a purely analytical contact detection algorithm, allowing for clinical usability and optimization of clinical outcomes.

## 1 Introduction

The patella plays a crucial role as a relay, acting as a pulley for the extensor system, which enhances the lever arm of the quadriceps and, consequently, boosts active extension strength in the knee joint. The patellofemoral joint is a part of the knee joint and refers to the articulation between the patella and the femur. It is a gliding joint that allows the patella to move smoothly along the groove at the lower end of the femur called the trochlear groove. The patella acts as a sesamoid bone, embedded within the quadriceps tendon, and plays a crucial role in the functioning of the knee joint. Poor patellar tracking can lead to increased contact pressures, patellar tilt, subluxation, or dislocation. The patellar trajectory refers to the path followed by the kneecap in relation to the femoral groove as the knee undergoes flexion and extension (Katchburian et al., 2003). Evaluating the patellar trajectory has been reliant on the surgeon’s subjective assessment, which involves direct visual observation during the surgical procedure (Best et al., 2020). Anatomical factors such as trochlear dysplasia, high-riding patella, ligament laxity, or an increased Q-angle can contribute to patellar instability. In some cases, surgical interventions may be required (Hayat et al., 2023). Total knee replacement (TKR) aims to alleviate pain, improve function, and enhance the overall quality of life for individuals with severe knee joint damage or degeneration (Innocenti et al., 2018). Following the placement of implants within the respective bones, the surgeon manually flexes and extends the knee of the anesthetized patient to witness the joint’s range of motion and assess the resulting patellar trajectory after the treatment has been applied. Despite advancements in implant design and surgical techniques for TKR, complications still arise, with around 10% of cases involving patellar issues (Putman et al., 2019) and these complications may require additional surgeries.

To address these challenges and improve treatment outcomes, there has been a growing interest in the use of musculoskeletal models and simulations in orthopedic surgeries (Fregly et al., 2012; Marra et al., 2015; Tischer et al., 2017; Van Rossom et al., 2019; Curreli et al., 2021). These tools enable objective predictions of post-treatment function and empower clinicians to explore different treatment options for patients. By utilizing these tools, the treatment planning process becomes more objective, allowing clinicians to tailor and optimize clinical outcomes according to the specific characteristics of each individual patient. Two main methods can be used for the definition of a mechanical system: Multibody Dynamics (MBD) and the Finite Element Method (FEM) (Gay Neto, 2023). In the Finite Element Method, each geometry is discretized into finite elements, forming a mesh with nodes that represents the physical properties of a mechanical system. Numerous FEM studies have examined the patellofemoral joint (Farrokhi et al., 2011; Aksahin et al., 2016; Islam et al., 2015). However, despite being a promising approach for knee osteoarthritis management, FEM time-intensive process (including pre-processing, processing, and post-processing) hinders its clinical usability (Paz et al., 2021). Consequently, FEM is presently confined to preoperative planning, focusing on aspects like the enhancement of implant design and surgical techniques, which are not subject to time constraints. Given this constraint and the anticipation of minimal elastic deformations in the involved bones, the exploration of the MBD approach emerged as a viable solution, providing a computationally efficient methodology to tackle clinical knee joint concerns (Bei and Fregly, 2004; Geier et al., 2015; Kebbach et al., 2020). This efficiency can potentially provide real-time biofeedback (Lugrís et al., 2023), rapid predictions of post-treatment function, or even time-reduced optimization of the surgical process to the surgeons, paving the way for its intraoperative application in the near future. Nonetheless, one significant computational challenge when integrating contact into a multibody dynamics framework revolves around collision detection (Li et al., 2023). It is challenging to implement methods and algorithms that can effectively and efficiently model the intricate phenomenon of contacting bodies with the necessary realism for MBD simulations. When the colliding bodies have complex 3D geometries, a general collision detection algorithm is required. A comparatively simple alternative is to approximate the freeform surfaces by discretized mesh elements and subsequently verifying proximity or overlap between them (Kebbach et al., 2020; Dopico et al., 2019). Although there are plenty of publications and software tools dealing with polygonal surfaces, in practice both the quality of polygonal surfaces and the efficiency of the tools can differ considerably (Hippmann, 2004). Therefore, when describing certain shapes, mathematical equations provide the optimal solution: both non-uniform rational B-spline (NURBS) surfaces (Bei and Fregly, 2004; Ateshian, 1993) and analytic formulation of 3D geometry (Dopico et al., 2019) have proven to be effective alternatives in previous applications. However, no work on MBD simulation was found that included a comparison of the patella’s movement and its forces with experimental results, primarily due to the invasive nature of these experiments.

In this work, due to negligible elastic deformations being expected in the involved bones and high-performance computing being sought, the simulation of the mechanical system was obtained through rigid-body multibody dynamics formulations. A comparison was made between two collision detection algorithms employed for the simulation of contact between rigid bodies: a mesh-to-mesh collision detection algorithm, which discretizes the bodies into triangular mesh surface elements, and an analytical contact algorithm, which uses analytical surface expressions to provide closed-form solutions for this contact problem. In addition, different 3D models of patellar and femoral implants were compared. Computational efficiency was considered, and histories of position, orientation, and pressure of the patella during the motion were compared with experimental measurements obtained from a sensorized 3D-printed test bench under various configurations. While this work does not include tibiofemoral contacts, the authors demonstrate that utilization of the patellofemoral digital twin already enables predictions for treatments addressing patellar stability issues when the tibiofemoral joint and the captured motions of femur and tibia are not affected. This is exemplified with a case study on tibial tuberosity transfer. Moreover, a new potential method for estimating tendon parameters from motion capture and simulation is introduced.

## 2 Material and methods

### 2.1 Movement and experimental data collection

To observe the patellar trajectory, two manual passive knee flexions and extensions were performed with the sensorized 3D-printed knee test rig described in (Michaud et al., 2023) which recreates a human leg, thus avoiding ethical issues. Bones were virtually cut and then 3D-printed (Prusa I3 MK3S, Prague, Czech Republic) to attach commercial tibia and femur implants (Microport^®^). Springs were used to recreate tendons. The movements of femur, tibia, and patella were obtained from the recorded trajectories of eight optical markers using 18 infrared cameras (OptiTrack FLEX 3, Natural Point, Corvallis, OR, United States) at a sampling frequency of 100 Hz. Additionally, spring tensions of femur and tibia were recorded using two tension load cells (RB-Phi-119, Phidgets, Calgary, Canada), and the contact force between the femur and the patellar prosthetic button was measured using a compact pressure load cell (FX29, TE Connectivity, Wört, Germany), also at a sampling frequency of 100 Hz. A second-order Butterworth filter with a cutoff frequency of 12 Hz was applied to the optically captured marker trajectories (Cuadrado et al., 2021), and a singular spectrum analysis (SSA) (Romero et al., 2015) with a window length of 30 was applied to the force measurements. Motion and force sensors allowed reproduction of the movement in the virtual model, fine-tuning of simulation parameters, and validation of experimental outcomes.

**FIGURE 1:**
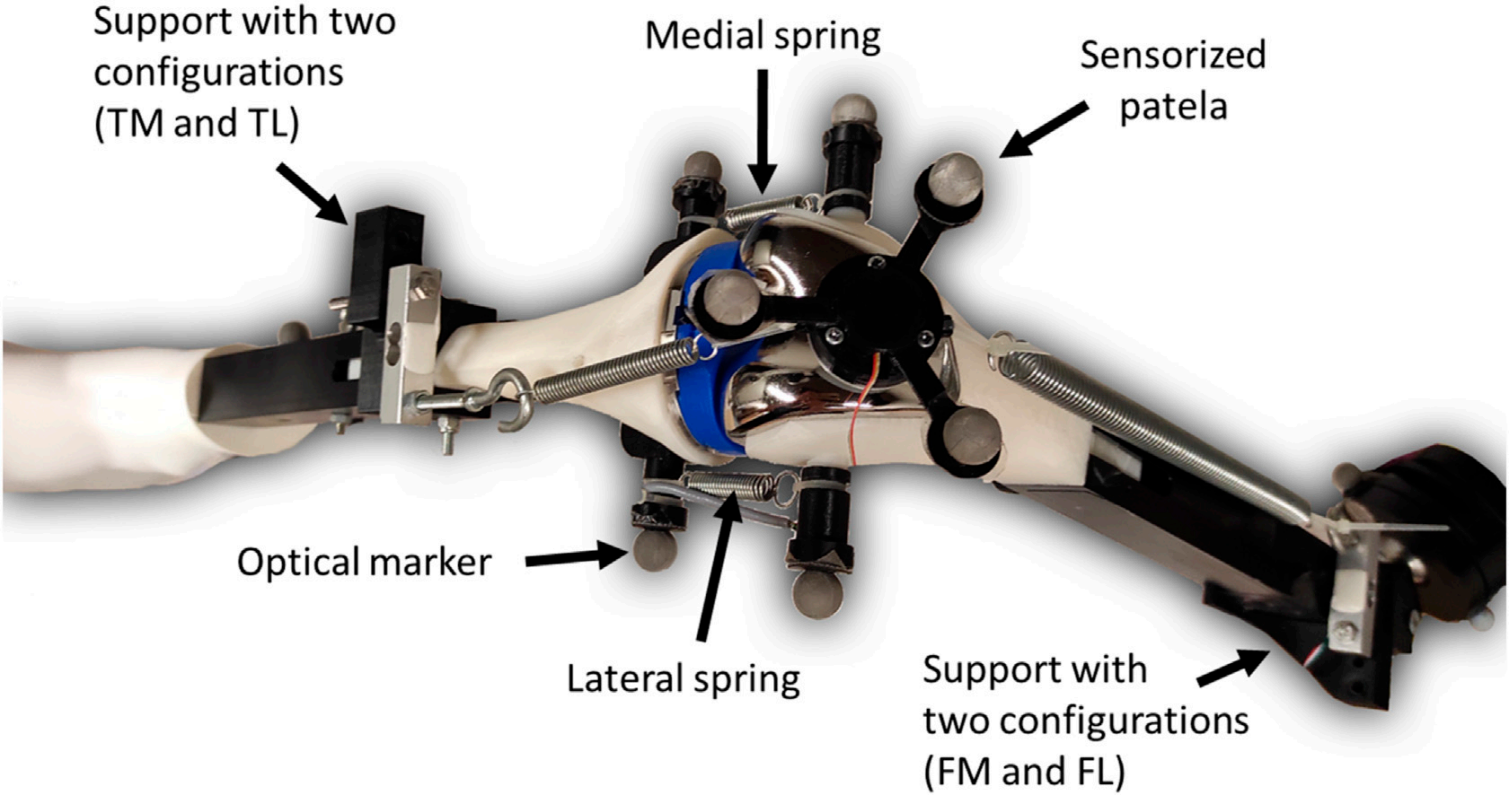
Modified sensorized knee test rig.

**FIGURE 2:**
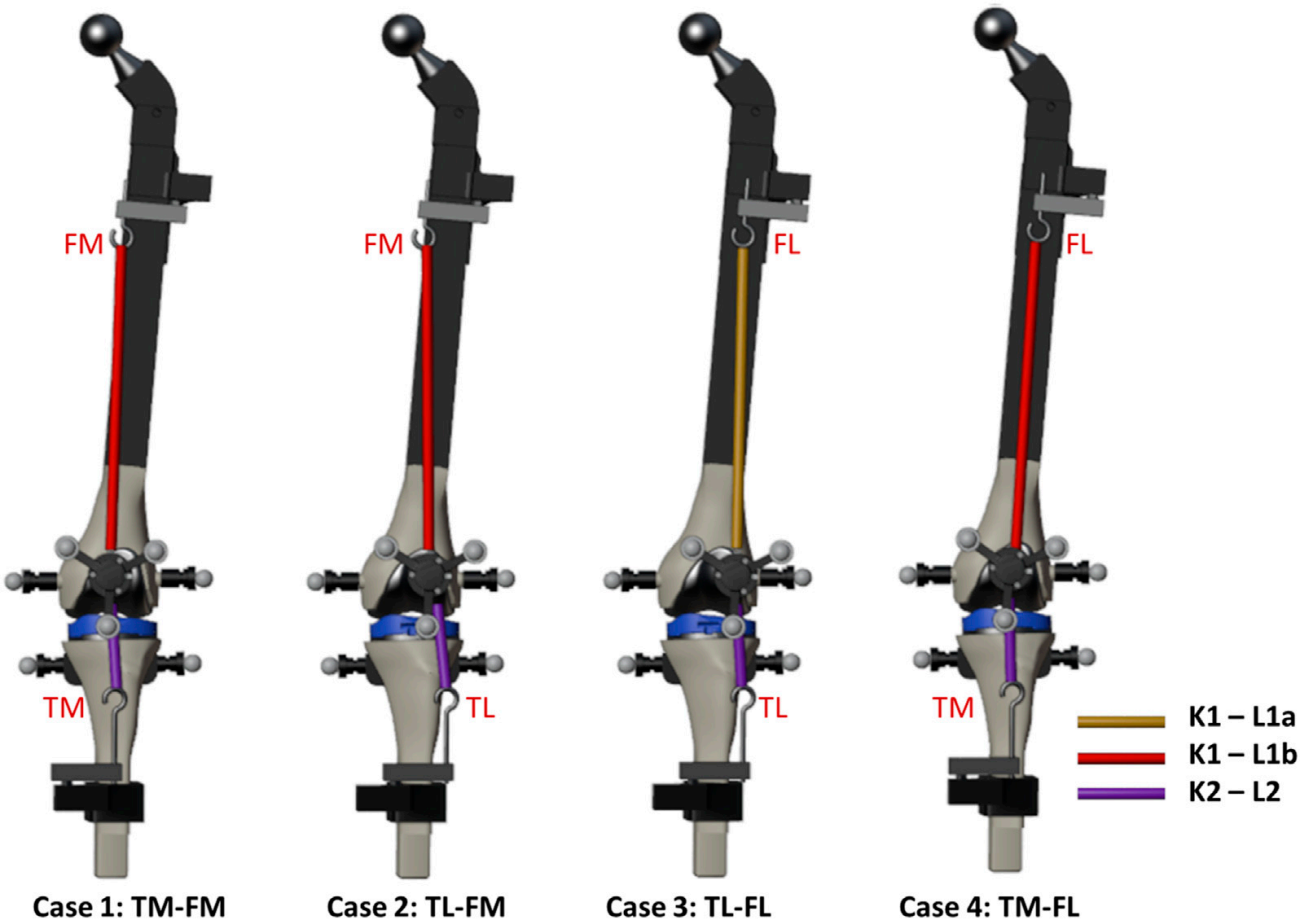
Knee anatomical configurations tested.

**FIGURE 3:**
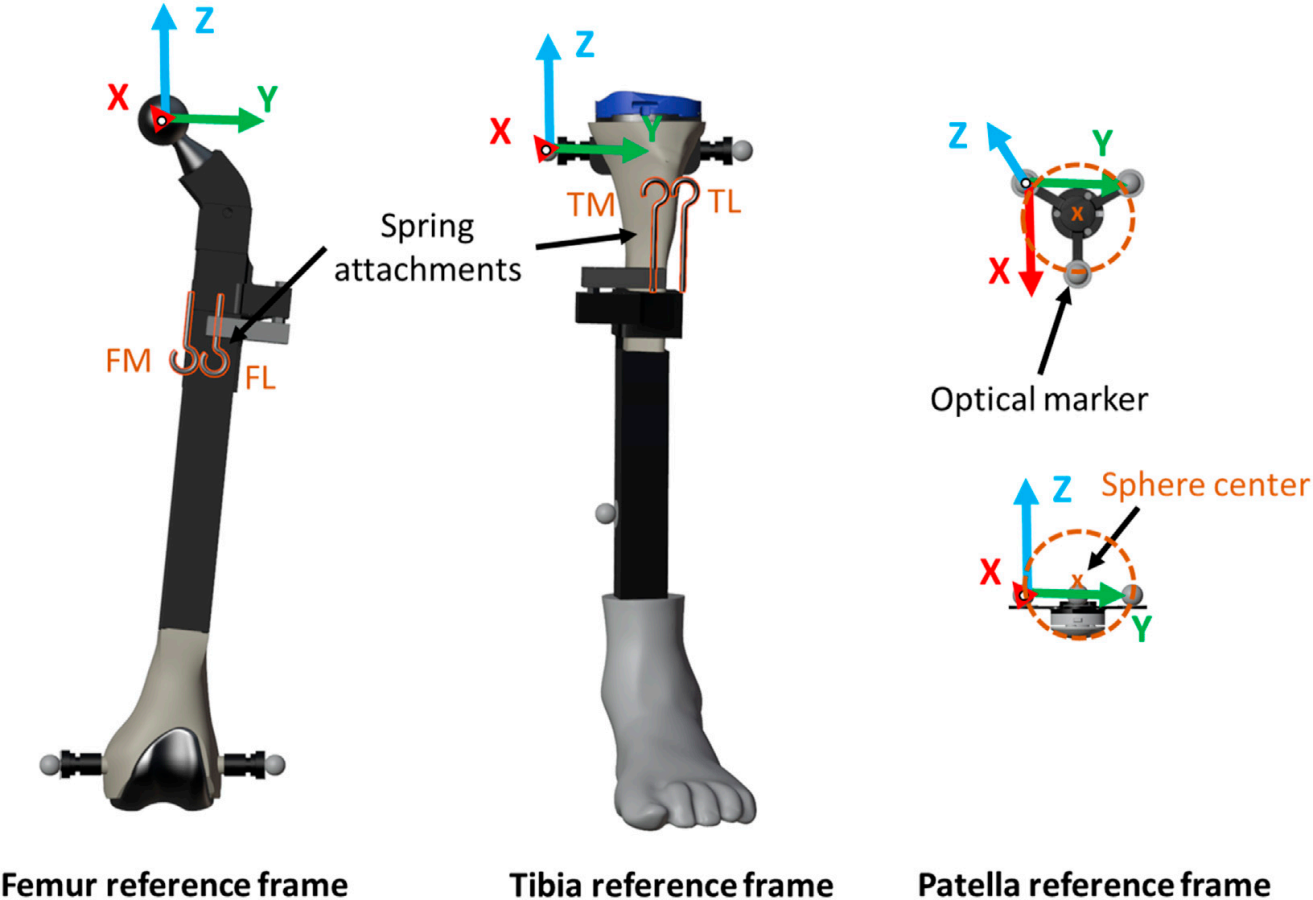
Computational model.

For this study, the knee test rig previously presented by the authors in (Michaud et al., 2023) was modified. The modifications included replacing the simplified hinge knee joint by springs on both sides to offer a more realistic representation of the knee joint. The tibia spring was short and stiff to simulate the patellar tendon (free length L2: 7.14 cm, stiffness K2: 629 N/m), while the femur spring was softer and longer to simulate the quadriceps tendon (free length L1a: 14.32 cm, stiffness K1: 156 N/m). In addition to the support that allows either a medial (FM) or a lateral (FL) attachment point on the femur, a support was also added to the tibia, enabling two configurations: tibia medial (TM) and tibia lateral (TL) (Figures 1–3).

The adjustable attachment points at tibia and femur, and the natural length of the femoral spring, were modified to simulate different knee anatomical configurations (Figure 1). These modifications correspond to altering the tendon laxity, the patellar height and the Q angle, also known as the quadriceps angle, which measures the alignment of the quadriceps muscles and the patella relative to the femur (Khasawneh et al., 2019). Femur and tibia supports provided two different positions each, resulting in a total of four different configurations (see Figure 2). Consequently, four distinct trajectories of the patella were tested. Additionally, the free length of the femoral spring was increased (case 3, Figure 2, L1b) by adding a rigid component of 1.96 cm to introduce an additional variation for simulation. Authors did not intend to reproduce any specific case, they simply wanted to validate their approach with different configurations that offer different patellar tracking to demonstrate that their approach allows to simulate any specific anatomical case.

The movement began with the leg flexed at approximately 45°, and was then flexed and extended twice. Cases 1 and 4 showed experimentally a normal tracking of the patella with different trajectories due to the different Q-angles. In contrast, in cases 2 and 3, a patellar dislocation occurred at complete extension during the experiments, so the leg was only partially extended in the first extension and extended until dislocation in the second extension. In addition, during the flexion of case 2, the patella did not engage with the femoral groove when the knee underwent flexion; instead, it got stuck in the superior part. This phenomenon is usually referred to as high-riding patella, also known as ‘patella alta’ (Tischer et al., 2017; Luyckx et al., 2009).

During TKR, tendon laxity, the patellar height and the Q angle can be corrected by adjusting the angles and heights of the cuts applied to the bones by the surgeon (Wang et al., 2010; Geier et al., 2015). Replicating this would necessitate 3D-printing multiple bones. However, since cases 1 and 2 share the same spring parameters, modifying the tendon attachment at the tibia in case 1 to address the patellar dislocation observed in case 2 can be considered a treatment for patellar issues. The corresponding surgical procedure is known as tibial tuberosity transfer (Clark et al., 2017). For this reason, the authors suggest using case 2 in section 2.6 as a pathological scenario, and case 1 as a potential treatment option (tibial tuberosity medialization) to simulate and validate predictions for a treatment, utilizing a single set of bones.

Due to the manually executed experimental actuation, the imposed motion was not identical for all configurations. The authors made every effort to reproduce the most similar motion; however, it is crucial to highlight that the purpose of this work was not to compare configurations against each other. The significance lies in the comparison between simulation and experimental results. Having different knee motions could be akin to observing different surgeons assessing the patellar trajectory. The recorded motion was used for the simulation, thus facilitating the comparison between simulated and experimental results.

### 2.2 Computational model

In this study, the leg model under consideration comprised three distinct rigid bodies: the femur, the assembly of tibia and foot, and the patella. The 3D geometries were identical to the physical components, including both the supports and the bones and implants. The femur remained fixed at the hip joint and capable of rotational movement around the three spatial directions. Tibia and patella were considered as two free bodies, each with its six degrees of freedom. This work focused on studying the interaction between patella and femur (using linear springs-dampers as tendons) and, more specifically, the contact of the patella with the femoral implant. The motion of femur and tibia was guided throughout this preliminary study.

The geometrical and physical parameters of the rigid bodies (local coordinates of points, inertias, etc.) were estimated from CAD models created in SolidWorks. These parameters, along with the mechanical constraints of the system, were then introduced into a custom-developed library (Dopico, 2016). The reference frame for the rigid bodies, as identified by the marker positions, is illustrated in Figure 3. The femoral body-fixed reference frame was defined by its “mechanical axis,” passing from the center of the knee joint to the center of the femoral head, and its medial-lateral axis, passing through the medial and lateral epicondyle (*Y*-axis). The sensorized patella body-fixed reference frame was defined by the patellar long axis (*X*-axis), the patellar medial-lateral axis (*Y*-axis, parallel to the femoral medial-lateral axis), and the patellar anterior-posterior axis (*Z*-axis) (Bull et al., 2002). The mechanical parameters of the springs were estimated from the experimentally recorded positions and forces. Utilizing Hooke’s Law to describe the spring force and Newton’s Law of Motion for the damping force (Sharma et al., 2019), the expression for the linear spring-damper force *F*_*i*_ of spring *i* is given by the formula:

$$F_{i}=K_{i}\left(L-L_{i}\right)-c_{i}*\dot{L}$$ (1)

Where *K*_*i*_ is the spring stiffness of spring *i*, *L*_*i*_ the natural length, *L* the displacement, $\dot{L}$ its velocity, and *c*_*i*_ = 0.01*K_*i*_ its damping coefficient.

### 2.3 Simulation

#### 2.3.1 Formulation

In this work, we utilized the ALI3-P formulation for the dynamics of the multibody system. This formulation, described in (Dopico et al., 2014), has undergone extensive development and evolution over the years, building upon the concepts presented in (Michaud et al., 2023; Cuadrado et al., 2021). The ALI3-P formulation is based on an Augmented Lagrangian approach, more specifically, an index 3 formulation in mixed coordinates (combining natural and relative coordinates). It incorporates velocity and acceleration projections on the constraint subspaces. For a comprehensive understanding of the equations of motion and the projections of velocities and accelerations, we direct the reader to reference (Dopico et al., 2019). The numerical integration was carried out by means of the Newmark integrator (Gavrea et al., 2005), with a time-step size of 1 ms.

#### 2.3.2 Guiding

The positions and orientations of the rigid bodies were determined based on the marker positions captured by the cameras of the motion capture system. To achieve this, the conventional methodology outlined by Vaughan (Vaughan et al., 1999) was applied, involving the following steps: (i) selection of three non-collinear entities, which could be markers or pre-defined joint locations, within each segment; (ii) establishment of an orthogonal reference frame for the corresponding segment using the selected entities; (iii) use of correlation equations to estimate the position and orientation of the rigid body.

**FIGURE 4:**
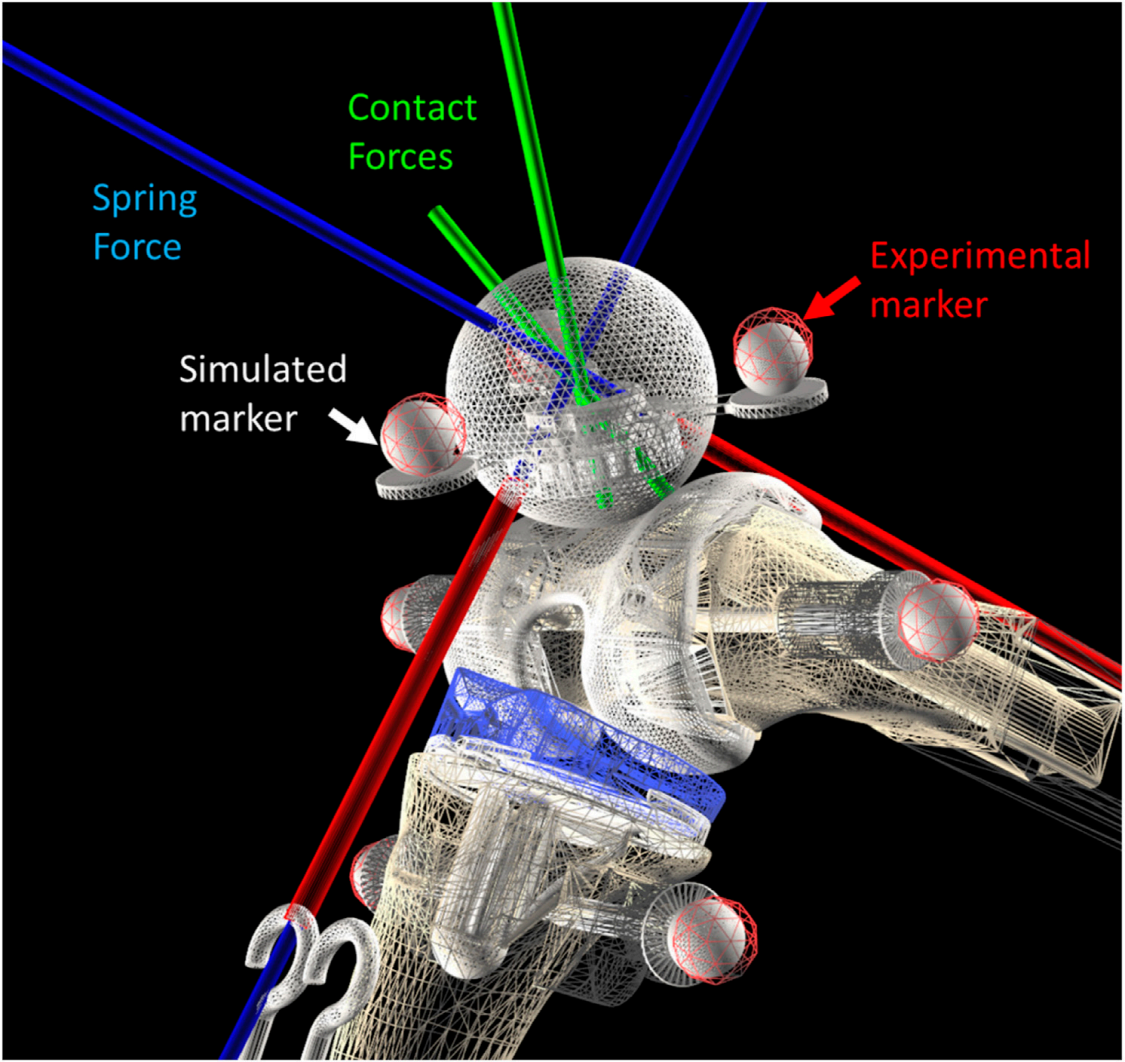
Mesh to mesh algorithm.

The optical motion capture system recorded the movements of femur and tibia, providing the inputs for the simulation. The simulation was guided by the experimentally measured values of all the degrees of freedom of the two rigid bodies. Additionally, the recorded movements of the patella were used for experimental validation of the simulation results (depicted by the red markers in Figure 4) and to approximate the patella to its initial static equilibrium position, which needed to be determined.

#### 2.3.3 Static equilibrium

To conduct a dynamic simulation of a multibody system, it is essential to acquire an initial set of positions and velocities that fulfill the constraint equations at configuration and velocity levels. In multibody systems with a static equilibrium configuration, it is advisable to initiate the simulation from it. This approach prevents the presence of initial high accelerations that could compromise the stability of the simulation. This, in turn, requires solving the static equilibrium equations of the system to determine the equilibrium configuration. Unfortunately, when the system involves bodies in contact, solving the static equilibrium problem becomes highly intricate, and, in some cases, multiple solutions may exist. For solving the nonlinear system, a Newton-Raphson iteration was used, similar to the one used to solve the equations of motion (Dopico et al., 2012).

#### 2.3.4 Contact model

Given that the contact area was lubricated in the experimental setup to mimic the synovial fluid function, the approach to consider the contact between the patella and the femoral implant was limited to the normal forces, excluding tangential forces (friction). The Flores model was selected for the normal force (Flores et al., 2011), its expression being:

$$F_{n}=k_{n}\delta^{p}\left(1+\frac{8\left(1-\varepsilon \right)}{5\varepsilon }\frac{\dot{\delta }}{{\dot{\delta }}_{0}}\right)n,$$ (2)

where $k_{n}$ is the equivalent contact stiffness, which depends on the shape and the material properties of the colliding bodies, *p* is the Hertz’s exponent, δ is the indentation and $\dot{\delta }$ its temporal derivative, ${\dot{\delta }}_{0}$ is the relative normal velocity between the colliding bodies when the contact is detected, ε is the coefficient of restitution, and **n** is the direction of the force. The subscript “n” comes from “normal”.

### 2.4 Contact detection

A major computational challenge when incorporating contact into a multibody dynamics framework lies in addressing collision detection. Successfully implementing methods and algorithms that can accurately and efficiently simulate the complex interplay between contacting bodies while maintaining the required realism for multibody system simulations is significantly challenging. Especially when dealing with colliding bodies possessing complex 3D geometries, the demand for a comprehensive collision detection algorithm is required. In this work, two contact detection approaches were implemented in the proprietary development library (Dopico, 2016) and compared.

#### 2.4.1 Mesh to mesh algorithm

**FIGURE 5:**
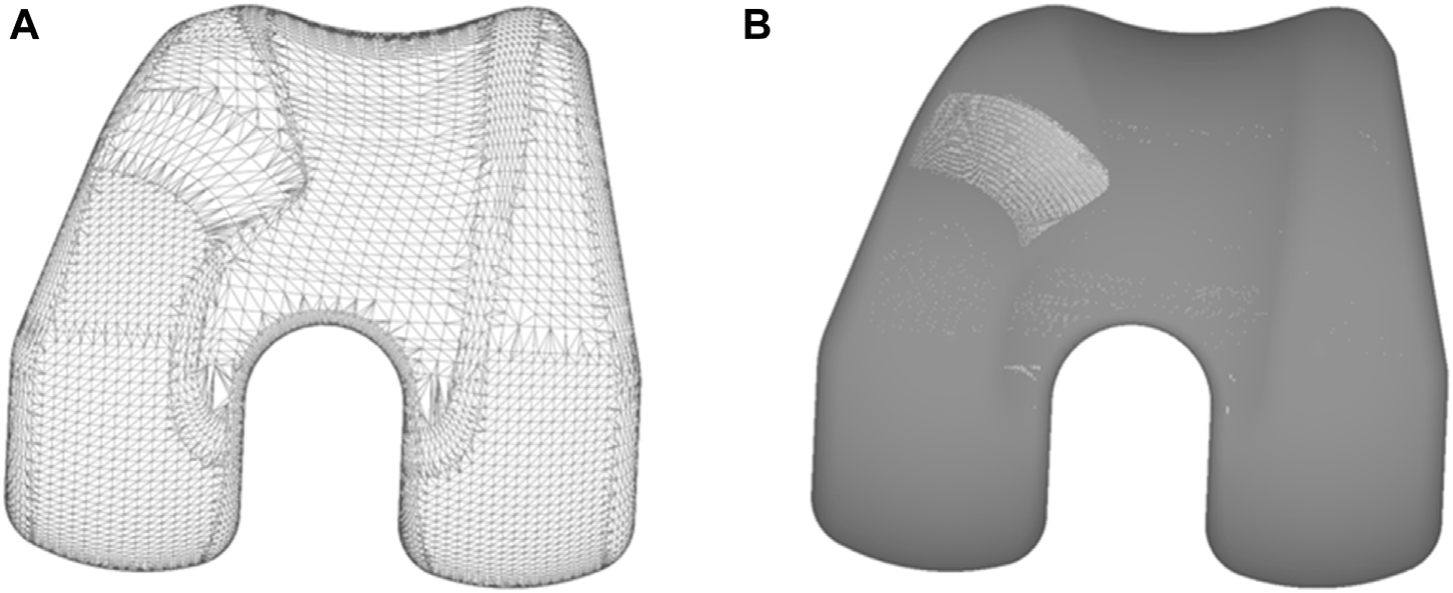
**(A)** Coarse mesh (CM); **(B)** Fine mesh (FM).

A comparatively simple alternative is to approximate the free-form surfaces by discretized mesh elements and subsequently verifying proximity or overlap between them. The triangular meshes of the colliding objects (femur and patellar button) were obtained in obj or stl formats from the native CAD files of the bodies. Due to potential differences in accuracy and efficiency of polygonal surfaces, two meshes were generated to compare these indicators. As shown in Figure 5, the finer mesh (FM, Figure 5B) had a tolerance deviation and angle of 0.006 mm and 0.5°, respectively, while the coarser mesh (CM, Figure 5A) displayed tolerances of 0.1 mm and 1°. A meshed icosphere (Figure 4) with similar mesh sizes was chosen over a meshed model of the patellar button because it possesses regular triangulation and avoids a point intersecting with numerous triangles at its apex.

The algorithm checks for penetration between the triangles and identifies corresponding contact points while calculating the maximum indentation. From this information, for each detected contact, it computes the normal forces (Figure 4, in green) employing the aforementioned contact models.

In order to speed up the collision detection and definition process, it is essential to check only the closest mesh elements. This efficiency is achieved through the utilization of an element classification structure known as the *Axis-Aligned Bounding Box Tree* (*AABB Tree*). The mesh undergoes a progressive subdivision and classification at each level into pairs of boxes, each approximately covering half of the volume of the previous box. This subdivision continues until the smaller *AABB*s exclusively enclose one element, typically a triangle.

During the collision characterization, the *AABB Tree* is compared against the patella object to determine if each *AABB* is in approximate contact with it. At every step, roughly half of the mesh elements are discarded. If the test is negative for a single *AABB*, its entire sub-tree can be discarded as well. This substantially reduces the number of checks to the order of log_2_(*N*) instead of *N* ^2^, where *N* is the number of mesh elements.

Once the list of contacting elements is identified, the contact contours are defined by their intersections. Subsequently, the averaged contact point, amount of penetration, and direction of the normal force for each contact can be computed from these contours. For a more detailed and comprehensive description of the algorithm, the reader is referred to (Dopico et al., 2019).

The time-step size for the CM had to be reduced to 0.1 ms for simulating cases 1 to 3 and further reduced to 0.05 ms for case 4.

#### 2.4.2 Analytic formulation

**FIGURE 6:**
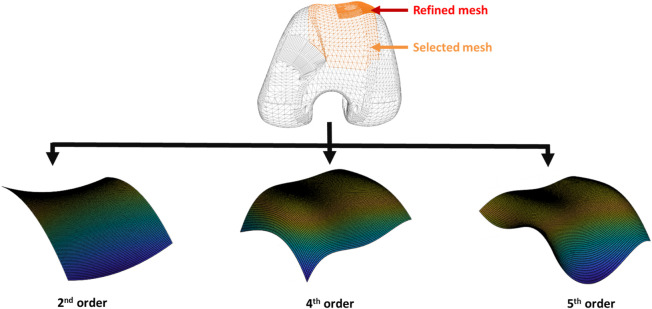
Polynomial equations derived from the vertex coordinates.

The analytic formulation involves employing mathematical equations to compute distances between the surfaces of geometric primitives. The specific equations used depend on the types of primitives being analyzed. In the scope of this study, only the interaction between the patellar button and the femoral implant was taken into account. The patellar button was represented by a spherical primitive, as only its spherical portion would come into contact with the femur. Similarly, for the femoral implant geometry, a comparable simplification was employed, concentrating solely on the surface that would interact with the patellar component (depicted in orange in Figure 6). Subsequently, a custom-made Matlab program was utilized to approximate this complex surface using a polynomial equation derived from the vertex coordinates. As illustrated in Figure 6, the mesh in the most critical area for patellar luxation was refined to enhance the accuracy of the approximation in this region. The geometry of the femoral implant was approximated using three polynomial equations: second, fourth, and fifth order (Figure 6), referred to as P2, P4, and P5, respectively, throughout the manuscript. This resulted in R-squared values of 91.54%, 99.36%, and 99,71%, respectively.

These equations take into account the position, orientation and size of the primitives. The objective is to ascertain whether two objects are in contact at a given time point. If the distance between the center of the sphere (representing the patellar button) and the femur surface is smaller than the radius of the sphere, it indicates the presence of a collision or contact. Based on this information and for each identified contact, the normal force is calculated (oriented perpendicular to the contact surfaces) utilizing the aforementioned contact model.

### 2.5 Experimental validation

To compare the results obtained from the computational simulations, the recorded experimental measurements were used as reference. The forces applied on the patella were validated by comparing the forces of the springs with the measurements from the load cells, and the contact force (only the normal component) with that obtained from the pressure load cell. The motion trajectory of the patella was also subjected to validation, achieved by contrasting the coordinates of the center of the patellar prosthetic button (sphere center, Figure 3) against those registered by the optical motion capture system. The latter was performed within the femur reference frame to avoid error accumulation (Figure 3). The tilt, flexion, and roll of the patella (rotations around the X, Y, and *Z*-axes, illustrated in Figure 3, respectively) were also compared with the experimental measurements. The error in the four tested knee anatomical configurations (Figure 2), utilizing the two described contact detection approaches, each of them with several resolution levels (CM, FM, P2, P4, and P5), was assessed by calculating the root mean square error (RMSE) between the respective pairs of data sets.

### 2.6 Estimation of tendon parameters from motion capture for treatment prediction

The authors employed well-established mechanical parameters of the springs to assess various approaches (CM, FM, P2, P4, and P5) presented in this work, thus avoiding the introduction of external errors. Following the evaluation, the authors recommend applying the most effective approach, P5 (selected based on accuracy and efficiency comparisons), to simulate a treatment prediction case under real conditions. Since the spring parameters (representing tendon parameters in the real context) are typically unknown, an optimization process was conducted to estimate them. This entailed utilizing the patellofemoral digital twin and conducting contact simulations to match the motion of the recorded patella of the pathological scenario (case 2). The measured forces were not used in this application, as it necessitates additional tools beyond the ones available in contemporary computer-assisted TKR surgeries that capture motion (Shatrov and Parker, 2020). The spring parameters were permitted to fluctuate within a range of 30% above and below their default values. The objective function was formulated as the summation of the RMSE of the distance error in the relative position of the patella. The genetic algorithm (*ga* function) provided by Matlab was employed to estimate the minimum value of this function.

In the current work, the utilization of the patellofemoral digital twin facilitates predictions for treatments that do not impact the tibiofemoral joint and the corresponding captured motion. As mentioned in subsection 2.1, this includes cases like the tibial tuberosity transfer, which entails modifying the tendon attachment at the tibia to address issues with patellar stability. As an example, in this study, the authors suggest using case 2 as a pathological scenario and case 1 as a potential treatment option (tibial tuberosity medialization). After optimizing the parameters with motion capture data from case 2, simulations for cases 1 and 2 were conducted using the novel optimal spring parameters. Experimental measurements from both cases were then used to assess the accuracy of simulating the pathological scenario and its potential treatment option.

### 2.7 Computational details

The calculations were conducted on a computer equipped with an Intel(R) Core(TM) i7-13700 KF @ 3.40 GHz processor, 32 GB RAM, and a 2 TB SSD running Windows 10 Pro. The analysis was performed using a single-threaded program written in Fortran 2008 and C++. The program was compiled using MSVC 2017 and Intel Fortran 2018. Efficiency was gauged by measuring runtime, distinguishing the time needed for obtaining the initial static equilibrium configuration and for executing the simulation.

## 3 Results

**FIGURE 7:**
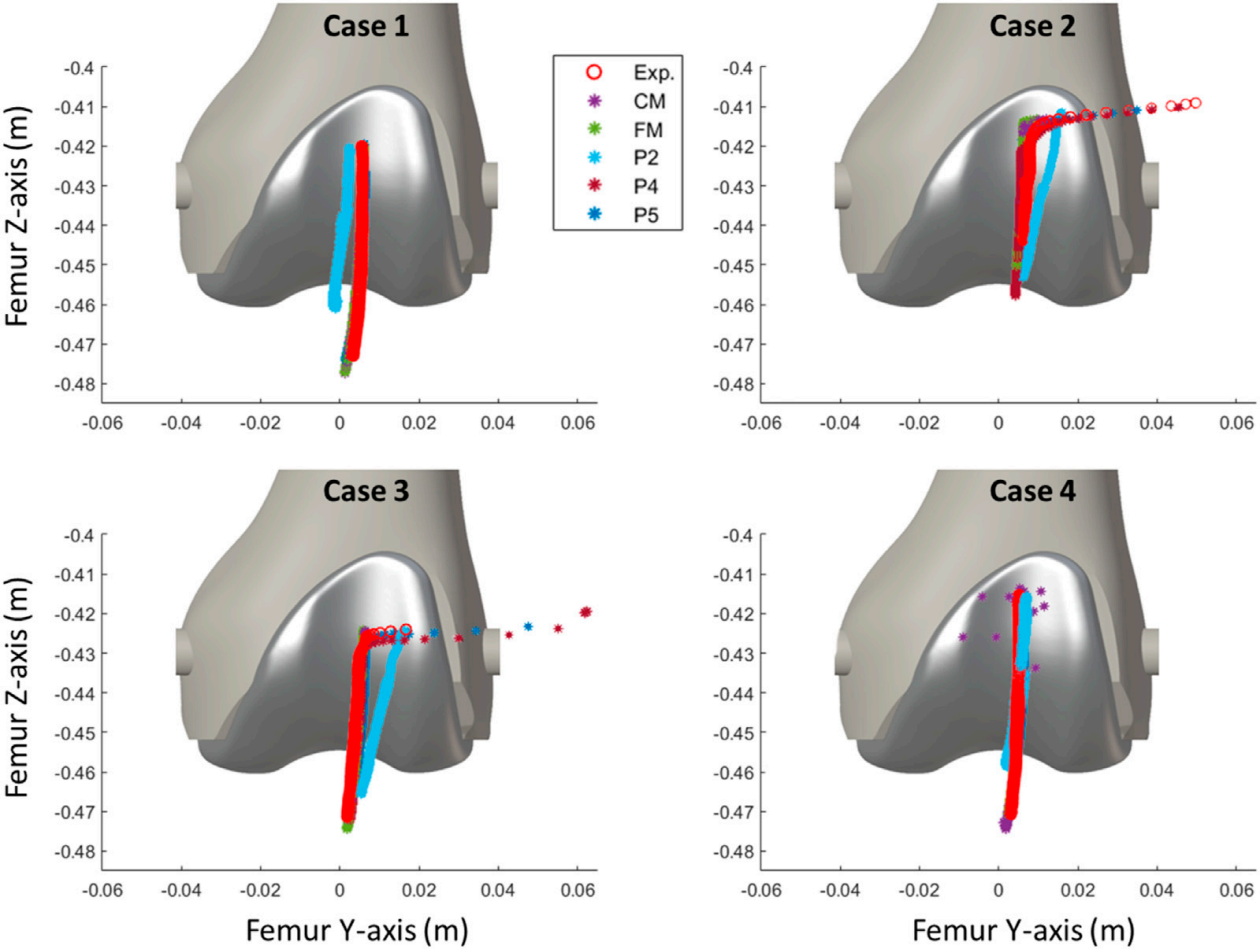
Comparison of the simulated patellar tracking using different contact detection algorithms.

**FIGURE 8:**
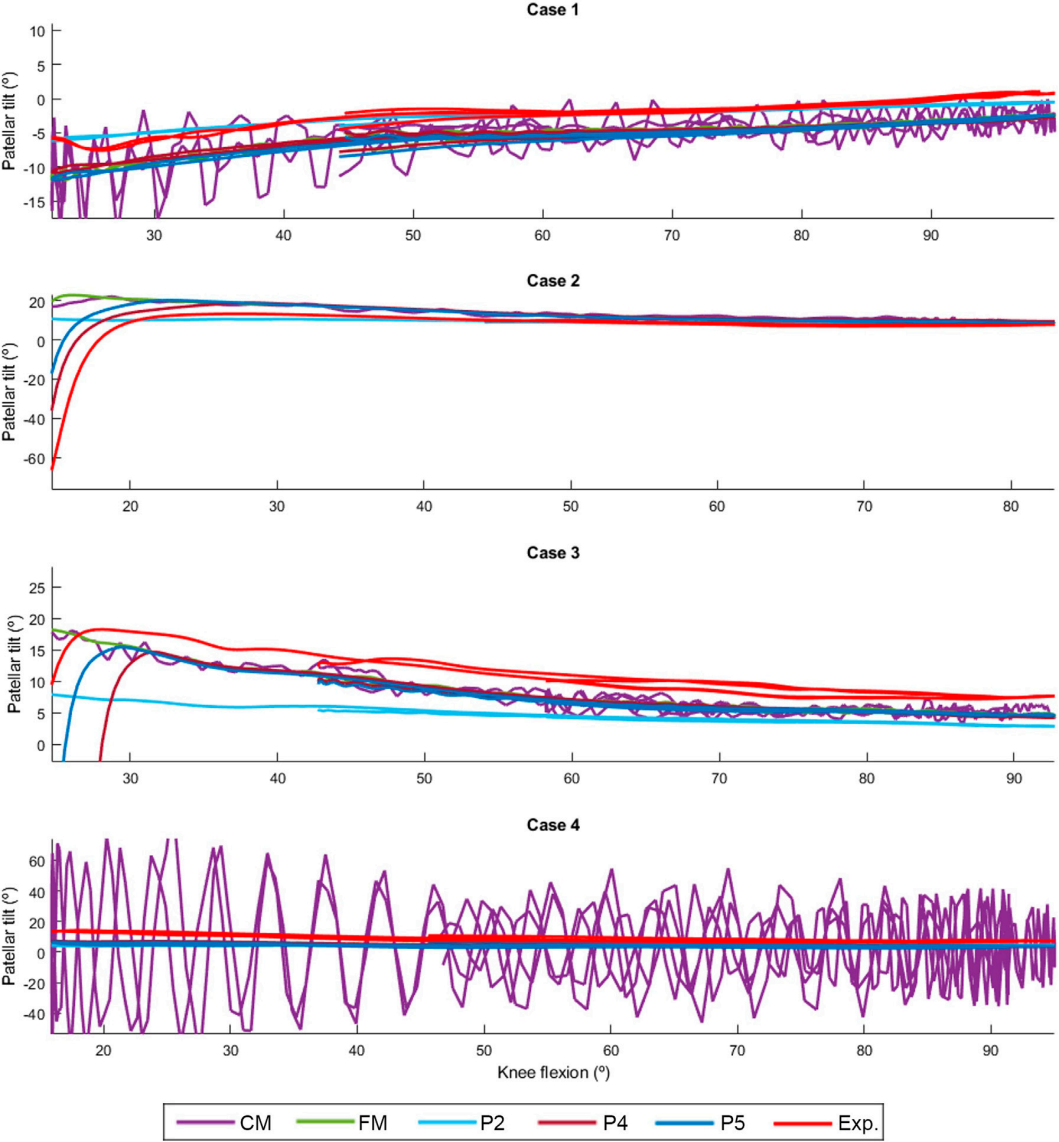
Comparison of the simulated patellar tilt using different contact detection algorithms.

The discrepancy in the four tested knee anatomical configurations (depicted in Figure 2), employing the two contact detection methods with different resolutions (CM, FM, P2, P4, and P5), was evaluated by computing the RMSE for each pair of corresponding data sets. The mean values of the four configurations by the different approaches are presented in Table 1. P2 yielded the least accurate force estimations and exhibited the highest position errors. CM exhibited notable discrepancies in contact force estimation and patellar orientation, attributed to substantial noise in the results despite reducing the time-step size. The trajectory shown in Figure 7 and the tilt angle shown in Figure 8 for case 4 with CM highlights the altered tilt of the patella due to the inaccurate contact force estimation. On the other hand, P5 exhibited the highest accuracy across all estimated values, with average errors of 2.12 N in force, 2.1° in orientation, and 1.5 mm in position, along all three axes. As highlighted in Figure 8, the primary orientation differences originated from the initial position and then remained constant throughout the knee motion. Besides FM, P5 was the only approach capable of closely replicating the observed high-riding patella phenomenon in case 2 (as depicted in Figures 7, 8), where the patella became wedged instead of sliding along the femur during flexion. Nevertheless, with the exception of P2, all approaches replicated the patellar luxations in cases 2 and 3, albeit some slightly offset in knee flexion (Figure 8).

**TABLE 1:** Accuracy and efficiency comparison of the different methods over the four cases (mean values, worst values in red, best values in bold).

		Mean values				
		Mesh to mesh algorithm		Analytic formulation		
		CM	FM	P2	P4	P5
**RMSE**	**Contact Force (N)**	**4.90**	3.44	4.06	3.38	**3.33**
	**Tibial Spring Force (N)**	1.52	1.39	**3.45**	1.30	**0.89**
	**Femoral Spring Force (N)**	1.96	2.01	**2.27**	**1.96**	2.15
	**Int. Rotation (°)**	**3.72**	0.65	**0.45**	0.74	0.59
	**Flexion (°)**	**2.47**	1.49	1.82	1.66	**1.38**
	**Tilt (°)**	**11.38**	4.85	4.46	6.60	**4.45**
	**X-coord. (mm)**	2.36	2.02	**4.53**	2.71	**1.44**
	**Y-coord. (mm)**	2.16	2.10	**3.67**	1.88	**1.36**
	**Z-coord. (mm)**	2.20	2.10	**5.81**	2.70	**1.70**
**Mean error**	**Distance (mm)**	2.98	2.78	**7.25**	2.99	**2.30**
**Initial configuration time (s)**		2.37	**33.29**	**0.51**	0.63	0.72
**Runtime (faster than real time)**		0.19	**0.07**	**6.00**	4.97	4.55

Regarding the efficiency of the various approaches, the mesh-to-mesh algorithm for contact detection is notably slower than the analytical formulation approach. Despite using a ten-times smaller time-step, the CM discretization was considerably faster than its more accurate counterpart, FM. Although increasing the polynomial order slightly extended the simulation runtimes, the most accurate P5 approach was still 4.55 times faster than real-time, or 2.57 times faster if the time required to obtain the initial equilibrium configuration is included.

This reduced computational time enables optimization using the contact simulation with the P5 approach. The optimization process took 1,233 s to provide an estimation of the spring parameters. Specifically, K1 and L1 were estimated with errors of 8% and 13%, respectively, while K2 and L2 had errors of 5% and 3%. Despite these discrepancies, the simulated pathological scenario and its respective potential treatment option were fairly reproduced (Table 2). Using the parameters optimized from motion captured data, the observed trajectories of the patella were accurately reproduced with mean position errors along all three axes of 1.56 and 1.65 mm, respectively. The high-riding patella and the dislocation were reproduced, and the improved trajectory resulting from tibial tuberosity medialization was also accurately predicted.

**TABLE 2:** Accuracy of the simulated pathological scenario (case 2) and its treatment (case 1) using estimated tendon parameters from motion capture.

		Pathological scenario	Treatment
**RMSE**	**Contact Force (N)**	2.56	3.62
	**Tibial Spring Force (N)**	1.12	1.16
	**Femoral Spring Force (N)**	2.45	2.51
	**Int. Rotation (°)**	0.61	0.67
	**Flexion (°)**	2.07	0.88
	**Tilt (°)**	3.63	5.44
	**X-coord. (mm)**	1.75	1.64
	**Y-coord. (mm)**	2.22	0.87
	**Z-coord. (mm)**	0.96	2.17
**Mean error**	**Distance (mm)**	1.66	2.61
**Initial configuration time (s)**		0.75	0.73
**Runtime (faster than real time)**		4.66	3.95

## 4 Discussion

In this study, aiming for computational efficiency and considering the expected negligible elastic deformations of the bones, the authors employed rigid-body multibody dynamics formulations to simulate the mechanical system. They conducted a comparative analysis between two collision detection algorithms used for simulating contact between rigid bodies: a mesh-to-mesh algorithm, which involves discretizing the surfaces of the bodies into triangular mesh elements, and an analytical algorithm utilizing analytical expressions of the surfaces to provide closed-form solutions for the contact problem. Furthermore, they compared various 3D model geometries of the femoral implant. As observed in (Dopico et al., 2019), the mesh-to-mesh algorithm induces artificial oscillations because the geometry is approximated using a set of triangles. This effect is particularly noticeable with coarse discretization, as the frequency and amplitude of the oscillations are related to the discretization size. Although the approach using the coarse mesh (CM) offered a reasonable approximation, it produced noisy results that require post-processing. Significantly reducing the mesh size almost eliminated the oscillations, but increased proportionally the computational time, thus impeding clinical usability.

The analytical contact detection techniques have proven to be a good alternative, offering reduced computational time. The simplest polynomial approximation (P2) was the fastest approach but also the least accurate. P4 and P5 showed variabilities of few millimeters in position with respect to experimental measurements that might stem from imperfections in 3D-printing, imprecisions in optical measurements magnified in the processing and body motion reconstruction steps, and inaccuracies in the analytical approximation of the femoral implant geometry. On the other hand, discrepancies in force could be attributed to small variabilities in the attachment points. The P5 polynomial approximation provided the best accuracy while maintaining a processing speed 4.5 times faster than real time. This makes it suitable for running optimizations to determine anatomical or treatment parameters and for conducting intraoperative simulations.

In this study, the authors proposed using experimental measurements of case 2 to represent a pathological scenario and those of case 1 as a potential treatment (tibial tuberosity medialization). Since the spring parameters (which mirror tendon characteristics in the real context) are typically unknown, an optimization procedure was carried out to estimate them from case 2 motion capture. Numerous local minima were encountered, complicating the optimization process and prolonging the search for the optimal solution. Nevertheless, the optimization time could certainly be decreased by employing more specialized optimization tools and strategies. As demonstrated in (Michaud et al., 2023), the mechanical system simulation allows for variations in spring parameters while maintaining the force equilibrium. Accurate reproduction of patellar tracking was achieved using the optimized parameters, despite minor deviations from the values of the initial calibration. This implies that the current state of the simulation permits the use of the patellofemoral digital twin to provide predictions for treatments addressing patellar stability issues that do not affect the tibiofemoral joint and the captured motions of femur and tibia, such as tibial tuberosity transfer or trochleoplasty (Nolan et al., 2018).

As a limitation, this preliminary work focused on the simplified load case of a passive knee flexion. This choice was made to facilitate validation through a low-cost sensorized 3D-printed knee test rig, while also addressing ethical considerations (Michaud et al., 2023). Despite the seemingly simple motion, this method has proven to be relevant for assessing patellar tracking during TKR surgeries (Best et al., 2020). Authors acknowledge that muscle activity and knee loads could potentially affect patellar tracking. Nevertheless, these parameters are not expected to impact the contact detection model presented in this study. Additionally, in their recent work, the authors demonstrated their ability to perform real-time inverse dynamics and estimate individual muscle forces, instilling confidence in maintaining efficient computational time (Lugrís et al., 2023).

In future studies, the authors will intend to employ more realistic tendon models instead of linear springs to validate the applicability of the proposed approach in real-life scenarios. They will also incorporate contact interactions between tibial and femoral implants, enabling predictions for TKR treatments. Nevertheless, this presents an additional challenge in accurately applying the corresponding forces to the virtual model, given the unclear force magnitude and the application points/surfaces associated with the surgeon’s maneuver. Lastly, in light of computational time performances, the authors are considering reconstructing the model motion, solving inverse dynamics and estimating contact forces, all while offering real-time visualization of the results (Lugrís et al., 2023).

## 5 Conclusion

The patellofemoral digital twin and contact detection algorithms developed in this study enable the reproduction of pathological scenarios resulting in patellar instability and facilitate the prediction of post-treatment function. Computational efficiency was taken into account, and the histories of position, orientation, and contact force of the patella during its motion were validated using experimental measurements obtained from a sensorized 3D-printed test bench. The best results were achieved through a purely analytical contact detection algorithm, allowing for clinical usability and optimization of clinical outcomes.

## Data Availability

The raw data supporting the conclusion of this article will be made available by the authors, without undue reservation.
